# Virtual telepathology in Egypt, applications of WSI in Cairo University

**DOI:** 10.1186/1746-1596-6-S1-S1

**Published:** 2011-03-30

**Authors:** Essam Ayad

**Affiliations:** 1 Department of Pathology. Faculty of Medicine, Cairo University & Italian Hospital in Cairo, Egypt

## Abstract

Telepathology, the practice of pathology at a long distance, has advanced continuously since 1986. Today, fourth-generation telepathology systems, so-called virtual slide telepathology systems, are being used for education applications. Both conventional and innovative surgical pathology diagnostic services are being designed and implemented as well. We have a successful experience in Egypt in applying the static & dynamic techniques in a pilot project between the Italian Hospital in Cairo (NPO) and the Civico Hospital in Palermo This project began in 2003 and continued till now. In 2004, centers in Venice, London and Pittsburgh participated actively in our project. During the past seven years we consulted on many problematic pathological cases with these different specialized pathological centers in Italy, UK & USA. In addition to the highly specialized scientific value of consulting on the cases and exchanging knowledge, we saved a lot of time and money and succeeded in providing our patients with a better medical service. In view of this success we have already established a new Digital Telepathology unit (DTU) in the pathology department, Cairo University, using the latest technique of telepathology which is Whole Slide Imaging (WSI) since one year. This unit is considered the first Digital pathology unit in all the universities of the whole Middle East. During the passed year we created a digital pathology library for the under graduate students using the WSI technique and changed the teaching method of the histopathology slides to be completely digital. We are building another digital pathology library (for post graduate candidates) which will be available to all pathology candidates in Egyptian universities & universities in the surrounding Arabic countries. We are also creating a digital pathology network between pathology centers in the Middle East for exchanging knowledge & telepathology.

## Background

Telepathology is the practice of pathology at a distance, viewing images on a monitor rather than directly through a light microscope [1]. In today's health care system, there are many uses of telepathology. For example, to "provide urgent services at sites either without a pathologist or sites with a pathologist requiring additional professional backup" Also, telepathology can "provide immediate access to sub-specialty pathology consultants" In general, telepathology techniques are classified into Static, Dynamic, Hybrid and Whole Slide Imaging (WSI) [2-4].

## Materials and method

**Figure 1:**
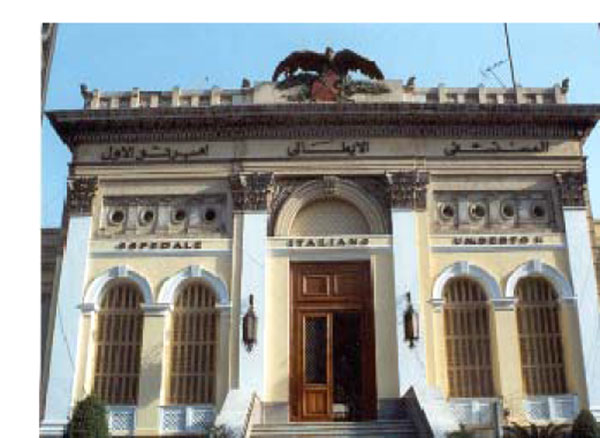
Italian hospital in Cairo

We have two successful experiences in Egypt in the application of the telepathology; the first was using the static and dynamic techniques of telepathology through a pilot project between the Italian Hospital in Cairo (NPO) and Civico Hospital in Palermo. (figure 1)

**Figure 2:**
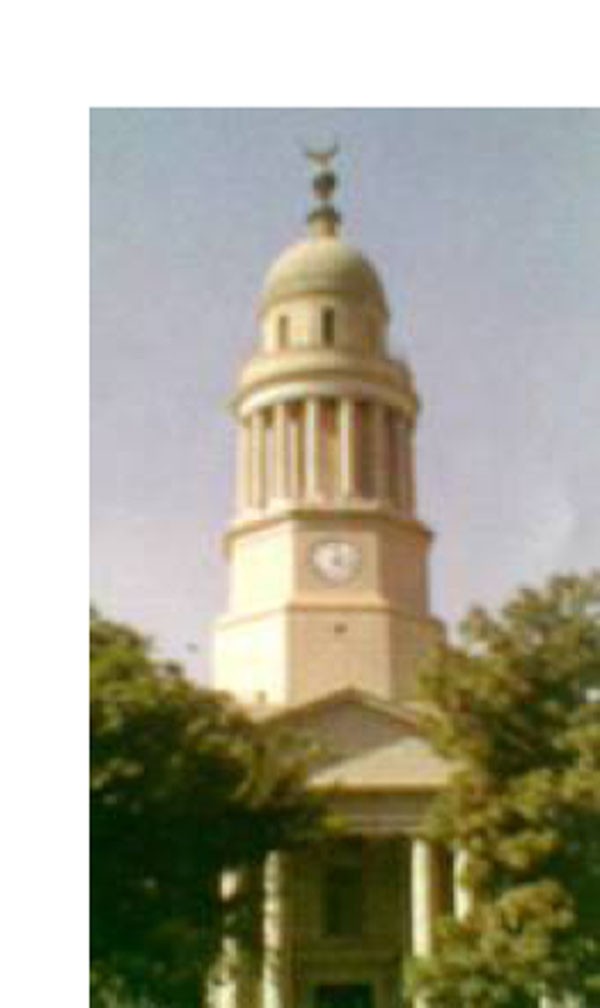
Kasr El-Eini Hospital, Cairo University

**Figure 3:**
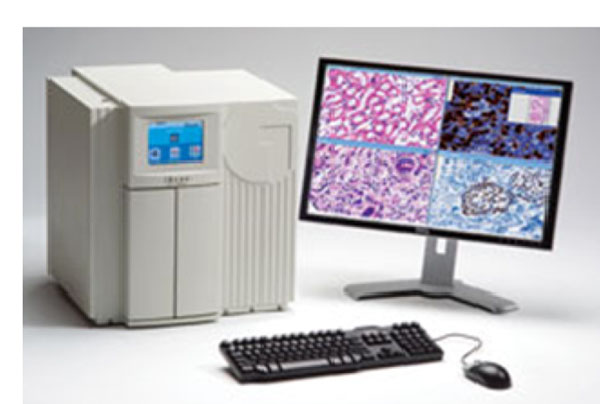
The Digital Pathology Unit in Pathology Department, Cairo University

This project began in 2003 by initiating the idea of the cooperation between CIVICO hospital in Palermo and the Italian hospital in Cairo, followed by the signing of a protocol of cooperation between both hospitals. The project is divided into four phases: Telepathology, Teleechocardiography, Tele-radiology & Tele-Endoscopy. The first phase (Telepathology) is now practically mature. The next phases of our project will be: Tele-echocardiography, Tele-radiology & Tele-endoscopy. For the First phase (Telepathology), these steps were taken: evaluation of the costs and securing the required funding; searching for the best instruments in the Egyptian market with the required functionality and certifications: downloading, and using in Cairo, the same software as used in Palermo; and the beginning of the connection [5]. In addition during the second year 2004), Ospedale S. Giovanni e Paolo Hospital in Venice, Charing Cross Hospital in London and University of Pittsburgh Medical Center Health System (UPMC) in USA joined as active participants in the telepathology project. The essential needs for our complete telepathology unit were straightforward: a suitable place, a server with a high-speed internet connection, a full computer system (computer, scanner & printer, a binocular microscope, a digital camera for gross pathology, a digital camera connected to the microscope to capture images from the microscope, a video-camera connected to the computer for video-conferences and suitable software for image processing and image transfer via the internet The benefits we expected and have achieved from the introduction of this telepathology unit are clear: better medical service, more distributed specialization, savings in time and money, increased knowledge exchange provides strong basis for improved teaching and learning practices. This unit has also provided its services to any pathology department in the Egyptian universities or any research centre. The success of the first project encouraged us to begin the second project which is establishing a new digital pathology unit in the pathology department, Cairo University (Figure 2) using the WSI technique [2-4]. (Figure 3)

This unit began work one year ago. During the passed year we created a digital pathology library for the under graduate students using the WSI technique and changed the teaching method of the histopathology slides to be completely digital. We are building another digital pathology library for post graduate candidates which will be available to all pathology candidates in Egyptian universities & universities in the surrounding Arabic countries. We are also creating a digital pathology network between pathology centers in the Middle East for exchanging knowledge & telepathology.

## Results

Over the past 7 years we consulted a lot of problematic pathological cases with these different specialized pathological centers in Italy, UK & USA. Beside the highly specialized scientific value of consulting the cases and exchanging knowledge, we saved a lot of time and money and offered our patients a better medical service. Even during the passed short period since the beginning of our digital pathology WSI unit, we changed the teaching method for the under graduate medical students to be completely digital which was markedly successful and widely accepted by the medical students. Using WSI technique in teaching also increased the acceptance of the entire staff member to use digitalized slides even in the routine examination of cases & telepathology. We also began scanning most of our valuable pathology slides to create a new digital pathology library for post graduate candidates which will be available to all pathology candidates in Egyptian universities & universities in the surrounding Arabic countries. We also began communication with pathology centers in the all Arabic countries within the Middle East to create a digital pathology network between for exchanging knowledge & telepathology.

## Conclusion

We concluded from our experience that telepathology is a very useful and applicable tool for additional consulting on difficult pathological cases. It has significantly increased knowledge exchange and thereby ensured our patients a better medical service, while simultaneously saving a lot of time and money over the previous practice [6]. In view of this success we established our Digital Telepathology Unit (DTU) in the pathology department, Cairo University. The application of WSI technique in teaching was greatly successful and encouraged us to create a huge digital pathology library which will expand our telepathology & E-learning programs to cover our staff and students both in Egypt and in the longer term in the wider Eastern Mediterranean.

## Competing interests

The authors declare that they have no competing interests.

## References

[B1] Digital imaging in pathology: the case for standardization J Telemed Telecare 2005 11 3 109 16 10.1258/1357633053688705 15901437

[B2] Use of whole slide imaging in surgical pathology quality assurance: design and pilot validation studies Hum Pathol 2006 37 3 322 31 10.1016/j.humpath.2005.11.005 16613327

[B3] Primary histologic diagnosis using automated whole slide imaging: a validation study BMC Clin Pathol 2006 6 4 10.1186/1472-6890-6-4 16643664 PMC1525169

[B4] Inter-hospital teleconsulting project between Cairo and Palermo Nuove Tecnologie in Medicina; Applicazioni Informatiche e telematiche in Medicina, Anno 4 2004 3-4 4 7

[B5] Overview of telepathology, virtual microscopy, and whole slide imaging: prospects for the future Hum Pathol 2009 40 8 1057 69 10.1016/j.humpath.2009.04.006 19552937

[B6] Medicine and humanities in the era of electronic information exchange, Anna Batistatou Diagnostic Pathology 2010 5 Suppl 1 S1 10.1186/1746-1596-5-S1-S1

